# Bariatric surgery patients in AUD treatment in Norway—an exploratory cross-sectional study

**DOI:** 10.1093/alcalc/agae007

**Published:** 2024-02-17

**Authors:** Jørgen G Bramness, Lars Lien, Jenny S Moe, Helge Toft, Susmita Pandey, Torgeir G Lid, Magnus Strømmen, John R Andersen, Ingeborg Bolstad

**Affiliations:** Department of Alcohol, Tobacco and Drugs, Norwegian Institute of Public Health, P.O.Box 222 Skøyen, 0213 Oslo, Norway; Norwegian National Advisory Unit on Concurrent Substance Abuse and Mental Health Disorders, Kjonerud kompetansesenter, Løvstadveien 7, 2312 Ottestad, Innlandet Hospital Trust, Brumunddal, Norway; Institute Clinical of Medicine, UiT The Arctic University of Norway, 9037 Tromsø, Norway; Norwegian National Advisory Unit on Concurrent Substance Abuse and Mental Health Disorders, Kjonerud kompetansesenter, Løvstadveien 7, 2312 Ottestad, Innlandet Hospital Trust, Brumunddal, Norway; Faculty of Social and Health Sciences, Inland Norway University of Applied Sciences, P.O.Box 400 Vestad, 2418 Elverum, Norway; Department of Alcohol, Tobacco and Drugs, Norwegian Institute of Public Health, P.O.Box 222 Skøyen, 0213 Oslo, Norway; Institute Clinical of Medicine, UiT The Arctic University of Norway, 9037 Tromsø, Norway; Faculty of Social and Health Sciences, Inland Norway University of Applied Sciences, P.O.Box 400 Vestad, 2418 Elverum, Norway; Norwegian National Advisory Unit on Concurrent Substance Abuse and Mental Health Disorders, Kjonerud kompetansesenter, Løvstadveien 7, 2312 Ottestad, Innlandet Hospital Trust, Brumunddal, Norway; Centre for Alcohol and Drug Research, Stavanger University Hospital, P.O. Box 8100, 4068 Stavanger, Norway; Faculty of Health Sciences, University of Stavanger, Telegrafdirektør Heftyes vei 73, 4021 Stavanger, Norway; Centre for Obesity Research, Clinic of Surgery, St. Olav’s University Hospital, Postboks 3250 Torgarden, 7006 Trondheim, Norway; Department of Clinical and Molecular Medicine, Norwegian University of Science and Technology, 7491 Trondheim, Norway; Department of Health and Caring Sciences, Faculty of Health and Social Sciences, Western Norway University of Applied Sciences, Svanehaugvegen 1, 6812 Førde, Norway; Førde Hospital Trust, P.O. Box 1000, 6807 Førde, Norway; Faculty of Social and Health Sciences, Inland Norway University of Applied Sciences, P.O.Box 400 Vestad, 2418 Elverum, Norway

**Keywords:** bariatric surgery, alcohol use disorder, treatment, clinical research

## Abstract

**Aims:**

Patients who have undergone some forms of bariatric surgery have increased risk of developing alcohol use disorder (AUD). In the present observational study, we compared patients with AUD who themselves reported to having undergone bariatric surgery with other patients in treatment for AUD.

**Materials:**

One-hundred-and-six consecutively enrolled patients in residential treatment for AUD were asked if they had undergone bariatric surgery. Sociodemographics, mental health-related, and alcohol use-related parameters were compared between those who had and those who had not undergone bariatric surgery.

**Results:**

Of the 106 patients with AUD, seven (6.6%; 95% confidence interval, 2.7%–13.1%) had undergone bariatric surgery. Six of seven patients had undergone such surgery were women (*P* < .001). The patients with AUD who had undergone bariatric surgery were similar to other patients with AUD on most other parameters, the exception being a larger number of alcohol units ingested to feel an effect of alcohol (adjusted odds ratio 7.1; 95% confidence interval 2.0–12.2; *P* = .007).

**Conclusion:**

The high number of patients with AUD that reported having undergone bariatric surgery emphasizes the risks following such a procedure. The overrepresentation of women may reflect than more women undergo such procedures. The unexpected finding that patients with AUD having undergone bariatric surgery seemed to need more alcohol to feel intoxicated warrants further research.

## Introduction

Several studies show that patients that have undergone at least some types of bariatric surgery have an increased risk for alcohol use disorder (AUD) (Ostlund  *et al.*  2013, Svensson  *et al.*  2013, King  *et al.*  2017, Strommen  *et al.*  2019). It has been speculated that this increased risk may be due to that one oral addiction is replaced by another (Mehr  2014), thus offering a psychological explanation for the increased prevalence of alcohol problems (Steffen  *et al.*  2015). The finding that AUD is much more prevalent after gastric bypass than after gastric banding and that binge-eating disorder does not seem to be a risk factor for AUD after surgery (Freire  *et al.*  2019), suggests an etiology in which there is a strong physiological component.

Studies show that heavy drinking is related to weight gain (Traversy  and  Chaput  2015). Some studies find that patients with obesity have an increased risk of AUD (Svensson  *et al.*  2023), while others find the opposite (Kleiner  *et al.*  2004). Some patients may have an AUD even before bariatric surgery (Ertelt  *et al.*  2008). However, most studies find that a substantial portion of the patients develop AUD after bariatric surgery (King  *et al.*  2012, Maciejewski  *et al.*  2020). A recent qualitative study on experiences of patients in specialized treatment for AUD with previous bariatric surgery supported this (Tvedt  *et al.*  2023). Risk factors for AUD in the general population include male gender (Gowin  *et al.*  2017), smoking (Grucza  and  Bierut  2006), impulsivity (Yang  *et al.*  2018), anxiety and major depression (Poikolainen  2000), and a family history of depression and other mental disorders, including AUD (MacDonald  *et al.*  1991, Lai  *et al.*  2022). Some of these are also risk factors for AUD after bariatric surgery together with obvious risk factors such as regular high alcohol consumption and previous AUD (Ivezaj  *et al.*  2019, Kanji  *et al.*  2019). Even in bariatric surgery patients, a positive family history of AUD is a predictor of AUD (Ivezaj  *et al.*  2014), reflecting a general hereditary component of AUD even in these patients (Deak  *et al.*  2019).

There is a gap in knowledge of AUD characteristics of patients treated with bariatric surgery. Characterization of the patients with AUD having undergone bariatric surgery, and comparisons with other patients with AUD, may help us tailor treatments more appropriate for this group (Dawson  *et al.*  2010). Few studies have done this. One study by Saules and co-workers found that 2%–6% of admissions to AUD treatment had undergone bariatric surgery, the number increasing (Saules  *et al.*  2010). Compared to other patients with AUD, bariatric surgery patients were more often women and more often non-smokers. The gender ratio may reflect that twice as many women than men are treated with bariatric surgery (Våge  *et al.*  2022). Saules and co-workers also found that bariatric patients with AUD drank larger quantities on heavy drinking days than other patients with AUD, a surprising fact considering the anatomical changes resulting in faster, higher, and longer duration of the blood alcohol exposure (Klockhoff  *et al.*  2002, Hagedorn  *et al.*  2007, Woodard  *et al.*  2011). On this basis, we wanted to increase the empirical knowledge in the field by (i) examining having undergone bariatric surgery and receiving treatment for AUD at three AUD treatment centers located in the south-eastern part of Norway and (ii) comparing clinical characteristic of a group of patients having undergone bariatric surgery with their fellow patients.

## Materials and methods

### Study participants

Data were collected in three rehabilitation clinics in the Eastern region of Norway from January 2018 to August 2019. The material has been described in a previous publication (Bolstad  *et al.*  2021). The clinics offer long-term residential treatment stays (>30 days) for people with substance use disorders (SUDs), where the majority have a diagnosis of AUD. People were considered for inclusion in the study if they had current AUD as diagnosed according to International Classification of Diseases 10th Revision (ICD-10) and were not in an unsuitable condition to participate in the study, as assessed by the clinical staff, due to severe somatic illness, psychosis, cognitive impairment, or inability to speak a Scandinavian language. Of the 366 patients who were admitted to treatment in the clinics during our inclusion period from January 2018 to March 2019, 224 (61%) were considered eligible for participation in this study. Eligible participants were provided with information about the study, and of these, 114 (51%) patients signed informed written consent and were enrolled in the study. Of the enrolled patients, 8 (7%) did not respond to the question of whether they had undergone bariatric surgery or not. Thus, 106 patients were included in the analyses, of whom 7 had undergone bariatric surgery*.* The study was approved by the Norwegian Regional Ethics Committee before data collection commenced (ID no: 21505/2017/1314).

### Measures

#### Background variables

Baseline data collection consisted of an interview asking about age (years), gender (male/female), educational level (below or above upper secondary education), marital status (living with a partner yes/no), and smoking status (current daily smoker yes/no). We collected biometric measures including waist circumference (cm), body mass index (BMI) calculated by weight (in kilograms) divided by the square of height (in meters), systolic and diastolic blood pressure (mmHg), and pulse (beats/minute) as well as data on psychiatric measures.

#### Psychiatric measures

The Mini International Neuropsychiatric Interview (M.I.N.I.) version 6.0 (Lecrubier  *et al.*  1997) was used to diagnose AUD and current anxiety disorders, including panic disorder, agoraphobia, social phobia, and generalized anxiety disorder. The Hopkins Symptom Checklist (HSCL-10) was used to measure psychological distress among the patients (Cloninger  *et al.*  1981). The 10 items were used to create an average score between 1 and 4, a higher score indicating more psychological distress. The level of depressive symptoms was measured using the BDI-II (Beck  *et al.*  1961, McPherson  and  Martin  2010). This self-report questionnaire consists of 21 questions asking the respondents how they have been feeling the preceding two weeks, with responses given on a 4-point Likert scale. Reponses are summed up into a total score ranging from 0 to 63, where higher score indicates higher level of depressive symptoms.

Patients were also asked about their exposure to trauma using a structured self-report form with five questions that previously have been used in a study of psychiatric inpatients (Toft  *et al.*  2018). The first three questions asked whether the person had experienced the following in his or her childhood: sexual assaults, physical abuse, and/or other traumatic events that subsequently caused significant problems (3). The last two questions dealt with experiences in adulthood: sexual assault or physical abuse (4) and other traumatic event that subsequently caused significant problems (5). We also collected information on ADHD symptoms using the Adult ADHD Self-Report Scale (ASRS) (Kessler  *et al.*  2005). The full version of this questionnaire consists of 18 items addressing inattentive or hyperactive–impulsive symptoms with five ordered response alternatives (0) “Never,” (1) “Seldom,” (2) “Sometimes,” (3) “Often,” and (4) “Very often.” A six-item screening version of the ASRS has demonstrated good specificity and sensitivity (Kessler  *et al.*  2005). We calculated the total score of these six items (Kessler  *et al.*  2007).

Lastly, we collected information on sleep using the Sleep Condition Indicator (SCI) (Espie  *et al.*  2014). The SCI has been developed to screen for insomnia and consists of eight questions including issues such as sleep onset delay; night-time awakenings; extent of the problem; and effect on daytime activities, mood, and relations. All items have response alternatives ranging from 0 to 4, where 0 indicates a poor state and 4 indicates no/little problems, resulting in a total sum score ranging from 0 to 32. A score of <16 indicates probable insomnia (Espie  *et al.*  2014). The SCI has been utilized in a previous publication by our group (Bolstad  *et al.*  2022).

#### Treatment drop-out

Patients who discontinued the treatment program and left the clinic before planned discharge were regarded as dropped out as opposed to patients who completed their stay or were still in treatment at 6 months follow-up.

#### Alcohol use disorder-related measures

We asked the patients about age of first drink (years), duration of drinking career (years), family history of alcohol problems (self-reported first-degree relatives with AUD), and experienced delirium tremens (self-reported). Alcohol use and symptom severity were measured with the Alcohol Use Disorders Identification Test (AUDIT), measuring both amount of drinking, binge drinking, harms of drinking, and consequences of drinking (Saunders  *et al.*  1993, Pradhan  *et al.*  2012). The severity of AUD was further evaluated using the Severity of Dependence Scale (SDS) measuring control over alcohol intake, preoccupation, and anxieties regarding drinking over the last year (Gossop  *et al.*  1995). The SDS was originally constructed to measure dependency of illicit drugs (Gossop  *et al.*  1997), but has later shown to also be a reliable and valid measure of alcohol dependence. The Norwegian version of SDS were used, for which Cronbach’s alpha ranging from 0.72 to 0.80 across a variety of substances has been reported (Cheng  *et al.*  2019, Kristoffersen  *et al.*  2019). Subjective effects of alcohol were measured at baseline using the instrument Self-Rating of the Effects of Alcohol (SRE), a 12-item questionnaire asking how many units a person had to drink to feel any effect, produce dizziness or slurred speech, be associated with stumbling or to have contributed to unintentionally falling asleep. Units required for each of these four experiences was registered for three different time points (Schuckit  *et al.*  1997): the first five times when the participant ever drank (SRE early), during the last period when drinking at least once a month for three consecutive months (SRE lately), and in periods of heaviest drinking (SRE heavy). We used the SRE lately measures.

#### Blood samples

Venous blood was drawn from the median cubital vein into serum tubes for analysis of phosphatidylethanol (PEth); carbohydrate-deficient transferrin (CDT); g-GT; ASAT; ALAT; ferritin; vitamins B12, B9, and D3; calcium; phosphate; prolactin; cholesterol; and C-reactive protein (CRP). The tubes were turned slowly upside down 8–10 times and left in a stand for 30 min before they were transferred to the laboratory for analysis.

### Statistical analyses

Descriptive statistics were used to assess sociodemographic data at baseline. A binomial exact method was utilized to determine the 95% confidence intervals (CI) for the percentage of patients who had undergone bariatric surgery. The precise proportion of the Norwegian population that has undergone bariatric surgery is unclear. Consequently, we extrapolated the prevalence using available historical data and official demographics. Assuming that there are 30 000 women and 10 000 men who have undergone bariatric surgery in Norway (personal communication with Villy Våge, director of The Norwegian national quality registry for bariatric surgery), within the age groups of 20–69 years (*n* = 3 485 915 in the overall population), this suggests that the prevalence is 1.76% (95% CI, 1.74%–1.78%) among women and 0.56% among men (95% CI, 0.55%–0.57%). We then employed these estimates, weighted for the ratio of men to women in the study sample, to calculate the anticipated prevalence. Due to the low N, we used Mann–Whitney U tests for the continuous variable and Fisher exact tests for the categorical variables. Logistic regression was employed to assess associations between various predictors of the alcohol use parameters found to be significant in the binary analysis. *P*-values were interpreted as continuous indicators of uncertainty and to avoid Type II statistical errors in the reporting of the results, we commented on data where the *P*-value was below .10. The statistical package SPSS was used for all analyses.

## Results

Of the 106 patients with AUD studied in this investigation, seven (6.6%; 95% CI, 2.7%–13.1%) reported having undergone bariatric surgery in contrast to an anticipated sex-adjusted prevalence of 0.84% (95% CI, 0.83%–0.85%) in the general population. Characteristics of these patients compared to those who had not undergone bariatric surgery are depicted in Table 1. Six of the seven patients who had undergone surgery were female (*P* < .001). They had a higher BMI (*P* = .003) and a tendency for higher waist circumference (*P* = .089) and higher diastolic blood pressure (*P* = .053). For most of the bariatric patients (*n* = 5), we had information on time of surgery making it possible to determine whether the self-reported drinking problem started before or after the surgery. For two patients, it started after surgery, and for the three others, it started before (data not shown in table).

**Table 1:** Sociodemographic characteristics, lifestyle, and health-related issues and mental health-related issues of the patients with SUD/AUD that have not or have undergone bariatric surgery. Some of the questions has less than 100% responders. All *P*-values below .1 are shown in bold.

		Undergone bariatric surgery?		
		No	Yes	
		*n* = 99 (93%)	*n* = 7 (7%)	*P*-value
Demographics				
Age (years)	Median (IQR)	52.9 (42.2–57.8)	49.5 (48.0–54.2)	.642 a
Sex (female)	*n* (%)	**19 (19)**	**6 (86)**	**<.001 b**
Educational level > upper secondary school	*n* (%)	20 (23)	1 (14)	.614 b
Marital status (living with a partner)	*n* (%)	16 (20)	2 (40)	.288 b
Lifestyle and physiology				
Smoking	*n* (%)	76 (77)	6 (85)	.677 b
Waist circumference (cm)	Median (IQR)	**101 (90–110)**	**105 (103–130)**	**.089 a**
BMI (kg/m^2^)	Median (IQR)	**26.1 (23.4–28.9)**	**32.0 (28.9–38.7)**	**.003 a**
Systolic blood pressure (mmHg)	Median (IQR)	127 (118–136)	137 (123–148)	.233 a
Diastolic blood pressure (mmHg)	Median (IQR)	79 (72–87)	92 (79–103)	.053 a
Pulse (beats per minute)	Median (IQR)	77 (67–85)	82 (77–87)	.250 a
Psychiatric comorbidities				
HSCL-10 score	Median (IQR)	**1.55 (1.30–2.03)**	**2.50 (2.40–2.60)**	**.025 a**
BDI-II score	Median (IQR)	15.5 (8.0–26.2)	22.0 (14.0–34.0)	.332 a
Life-time suicide attempt	*n* (%)	27 (30)	2 (29)	.968 b
Anxiety disorder	*n* (%)	26 (29)	1 (14)	.623 b
ASRS score	Median (IQR)	33 (24–44)	37 (32–57)	.176 a
Childhood trauma experience	*n* (%)	47 (68)	5 (100)	.132 b
Adulthood trauma experience	*n* (%)	**42 (61)**	**5 (100)**	**.079 b**
Life quality and sleep				
Quality of life (higher is worse)	Median (IQR)	14 (11–17)	14 (12–17)	.759 a
Fatigue severity scale	Median (IQR)	46 (41–55)	59 (37–62)	.262 a
Sleep condition	Median (IQR)	18 (13–28)	14 (8–22)	.318 a
Dropped out of treatment	*n* (%)	29 (30)	1 (14)	.371 b

*P*-values below .1 are indicated in bold. BMI: body mass index calculated from height (in meters) and weight (in kilograms) after the formula m/kg^2^; HSCL: Hopkins Symptom Checklist; BDI-II: Beck Depression Inventory 2; ASRS: Adult ADHD Symptom Rating scale. Descriptive statistics given as medians [IQR (interquartile range): 25th and 75th percentile] and group differences tested with Mann–Whitney U test for continuous variables (a) and given as frequencies and percent and tested with Fisher exact test for categorical variables (b).

Patients with AUD having undergone bariatric surgery scored higher on HSCL-10 compared to other patients with AUD (*P* = .025). In multivariate analysis (data not shown in table), this did not withstand adjustment for gender. There was a tendency to report more adult trauma (*P* = .079), but without differences in BDI-II score, anxiety disorder, ASRS score, childhood trauma experience, or life-time suicide attempt. Neither were there any differences in fatigue, quality of life, sleep problems, or drop out.

The patients with AUD who had undergone bariatric surgery reported needing more drinks to feel any effect of alcohol compared to other patients with AUD (*P* = .048) and there was a tendency for these patients to report a higher severity of dependence score (*P* = .067) (Table 2). In addition, a broad range of alcohol use parameters were measured. We found no difference between patients with or without bariatric surgery in AUDIT score, days since last drink, amount at last drink, age of first drink, drinking career duration, family history of alcohol problems, or experience with delirium tremens, nor were there any differences in a broad range of biochemical measures related to alcohol use (Table 3). The number of standard units of alcohol before noticing any effect was significantly higher among those who had undergone bariatric surgery (median (IQR)); 6 (4–8) vs. 8 (6–21), (*P* = .048). This difference withstood adjustment for gender, BMI, HSCL-10 score, and SDS score (Supplementary Table S2) (*P* = .007). The tendency for a higher severity of dependence among those having undergone bariatric surgery did not withstand adjustment for gender (data not shown in table).

**Table 2:** Alcohol-related measures of the patients with AUD that have not or have undergone bariatric surgery. All *P*-values below .1 are shown in bold.

		Undergone bariatric surgery?		
		No	Yes	
		*n* = 99 (93%)	*n* = 7 (7%)	*P*-value
Substance use-related measures				
AUDIT	Median (IQR)	28 (21–34)	28 (23–33)	.910 a
Severity of Dependence score	Median (IQR)	**10 (7–11)**	**13 (10–14)**	**.067 a**
Days since last drink	Median (IQR)	19 (13–30)	17 (11–31)	.753 a
Amount at last drink (number)	Median (IQR)	13 (8–22)	15 (12–30)	.539 a
Amount at last/days since	Median (IQR)	0.72 (0.32–1.20)	0.88 (0.42–1.77)	.494 a
Age of first drink	Median (IQR)	15 (13–16)	15 (14–16)	.994 a
Drinking career duration (years)	Median (IQR)	15 (7–22)	11 (9–18)	.493 a
Drinks to feel any effect lately	Median (IQR)	**6 (4–8)**	**8 (6–21)**	**.048 a**
Drinks to dizzy/slurred speech lately	Median (IQR)	9 (6–12)	10 (6–36)	.602 a
Family history of alcohol problems	*n* (%)	58 (65)	4 (67)	.941 b
Experienced delirium tremens	*n* (%)	20 (20)	2 (29)	.598 b

*P*-values below .1 are indicated in bold. Descriptive statistics given as medians (IQR (interquartile range): 25th and 75th percentile) and group differences tested with Mann–Whitney U test for continuous variables (a) and given as frequencies and percent and tested with Fisher exact test for categorical variables (b).

**Table 3:** Blood sample measurements of the patients with AUD that have not or have undergone bariatric surgery. Data are given as median and interquartile range. Significance testing was done by Mann–Whitney U test. All *P*-values below .1 are shown in bold.

	Undergone bariatric surgery?		
	No	Yes	
	*n* = 99 (93%)	*n* = 7 (7%)	*P*-value
Blood values			
PEth	0.22 (0.07–0.57)	0.20 (0.11–0.47)	.995
CDT	0.8 (0.5–1.0)	0.8 (0.6–1.3)	.749
g-GT	33 (23–79)	36 (32–52)	.525
ASAT	24 (19–37)	22 (20–35)	.947
ALAT	32 (20–47)	33 (23–60)	.563
ASAT/ALAT	0.82 (0.63–1.04)	0.74 (0.65–0.91)	.607
Ferritin	**106 (59–188)**	**18 (11–36)**	**<.001**
Vitamin B12	389 (303–463)	436 (302–693)	.475
Vitamin B9	17 (12–27)	33 (9–43)	.134
Vitamin D3	66 (45–84)	60 (57–75)	.908
Calcium	2.36 (2.28–2.40)	2.33 (2.24–2.37)	.373
Phosphate	1.14 (1.04–1.26)	1.10 (0.82–1.41)	.749
Prolactin	138 (96–191)	143 (121–242)	.296
CRP	1.0 (1.0–4.0)	2.0 (1.5–7.5)	.827
Cholesterol	**5.0 (4.6–6.0)**	**4.2 (4.0–4.3)**	**.005**

*P*-values below .1 are indicated in bold.

The AUD patients having undergone bariatric surgery had a much lower ferritin level measured in their blood compared to other patients with AUD (*P* < .001) (Table 3).

As the large majority of those having undergone bariatric surgery were female, we repeated all the analyses in Tables 1–3 only for females only. In Supplementary Table S2, the results from this analysis, including the significant differences mentioned above, are shown for females only.

## Discussion

The present small-scale explorative study is one of the first to compare patients with AUD who had undergone bariatric surgery with other patients with AUD. In this naturalistic sample of patients with AUD, there was quite a high share of patients who had undergone bariatric surgery. These patients were more often female. Most strikingly, these patients did not differ from other patients with AUD on most measured parameters, the major exception being the amount of alcohol needed to feel intoxicated, which was significantly higher among those having undergone bariatric surgery. Some lifestyle- and surgery-related measures like weight and iron status differed.

A 6.6% share of the patients with AUD having undergone bariatric surgery was relatively high, higher than in some other studies (Wiedemann  *et al.*  2013), but similar to what is found by others (Saules  *et al.*  2010). A rate of 6.6% among the patient group compared to the 0.84% in the general population gives a ratio comparable to the hazard ration of 7.2 found in a Danish national registry study of bariatric surgery patients (Bramming  *et al.*  2021). Furthermore, official statistics indicate that one-third of all patients with AUD treated in specialized health care are female, with a little less in residential treatment, the setting we are investigating here (Helsedirektoratet  2017). In this study 24% of the patients were female, which is comparable to the national figures. However, six of the seven patients with AUD that had undergone bariatric surgery were female. Earlier Norwegian studies indicate that 70% of the patients who undergo bariatric surgery are female (Jakobsen  *et al.*  2010). Even with such a gender bias, the female overrepresentation are remarkable, but resembles what is seen in the Danish study (Bramming  *et al.*  2021).

Most alcohol use parameters investigated in the current study did not differ between those having undergone bariatric surgery and the other patients. The similar measures included AUDIT score, days since last drink, amount at last drinking day, age at first drink, drinking career duration, and family history of alcohol problems. There was seemingly a difference in SDS score, but this disappeared when controlling for other factors. Only a few other studies have investigated such characteristics finding the same for AUDIT score (Wiedemann  *et al.*  2013) and amount alcohol taken at last drinking day (Saules  *et al.*  2010).

We did, however, find a difference in reported number of units of alcohol needed to feel an alcohol effect. Here, and rather surprisingly, we found that those having undergone bariatric surgery reported having to drink more before feeling any effect. This has been observed in other empirically based studies (Saules  *et al.*  2010) and is also known from clinical experience. The surprise lies in the knowledge that alcohol is absorbed faster and more in those having undergone bariatric surgery (Steffen  *et al.*  2013, Pepino  *et al.*  2015, Acevedo  *et al.*  2020), thus leaving them more prone to the effects of alcohol. Consequently, one would expect that they needed less alcohol to feel an effect, not more, as we have observed in this study. However, this finding is not viewed in relation to their preoperative alcohol use and might be a consequence of other things like body composition despite the finding withstanding adjustment for BMI.

Even if there is a wide normal range for ferritin levels the low levels in those having undergone bariatric surgery points to a known risk after such procedures (Sandvik  *et al.*  2021). The rather high levels of vitamins indicates that patients with AUD in treatment are given adequate supplements of these, but sufficient iron supplements should also be given (Shipton  *et al.*  2021).

This was a small study with few patients and possibly underpowered to identify real differences and thus open to spurious findings. The study must be considered an exploratory one, and results should be interpreted with caution. The study was designed for another purpose, but with collection of data on several life events including bariatric surgery. It is a shortcoming that we did not know what kind of surgery they have undergone and relied on self-report of such surgery, not official registration. Some may have had gastric sleeve, and some may have undergone gastric bypass, the former with probably greater risk of AUD (Azam  *et al.*  2018). Lastly, we based our conclusion on AUD followed or preceded surgery on self-report. Our sparse data indicate that both scenarios may have been present.

In conclusion, the present study indicated that patients having undergone bariatric surgery were overrepresented in AUD treatment centers. In the present study, almost all the patients having undergone bariatric surgery were women. On most parameters, these patients resembled other patients with AUD, except that they needed more drinks to feel an effect of alcohol. More studies on the characteristics of bariatric surgery patients that have developed AUD are needed to improve the knowledge of both risk factors for AUD among patients having undergone bariatric surgery and to identify if these patients have special needs regarding treatment of their AUD.

## Supplementary Material

Supplementary_tables_ALC_23_0141_agae007

## Data Availability

The data are only available after reasonable request to the corresponding author and an application to and approval by the ethics committee that approved the study.
