# Supplementary material for: Bariatric surgery patients in AUD treatment in Norway—an exploratory cross-sectional study
Source: Alcohol Alcohol. 2024 Feb 17;59(2):agae007. doi: 10.1093/alcalc/agae007 (PMC11445783; doi:10.1093/alcalc/agae007)
Supplement: Supplementary_tables_ALC_23_0141_agae007 [file supplementary_tables_alc_23_0141_agae007.docx]

**Supplementary table S1.** Some lifestyle and health related issues, mental health and alcohol and substance use related issues of *female* patients with SUD/AUD that have not or have undergone bariatric surgery. Some of the questions has less than 100 % responders. All p-values below 0.1 are shown in bold.

|  |  | **Undergone bariatric surgery?** | |  |
| --- | --- | --- | --- | --- |
|  |  | **No** | **Yes** |  |
|  |  | n = 19 (76%) | n = 6 (24%) | **p-value** |
| **Lifestyle and physiology** |  |  |  |  |
| Smoking | n (%) | 11 (58) | 6 (100) | 0.156 b |
| Waist circumference (cm) | Median (IQR) | **62 (83-105)** | **105 (102-111)** | **0.036 a** |
| BMI (kg/m^2^) | Median (IQR) | **25.6 (22.4-28.5)** | **30.7 (28.3-38.0)** | **0.007 a** |
| **Psychiatric comorbidities** |  |  |  |  |
| HSCL-10 score | Median (IQR) | 2.30 (1.85-3.20) | 2.60 (2.40-) | 0.645 a |
| BDI-II score | Median (IQR) | 22.0 (13.8-31.5) | 22.0 (12.0-) | 0.959 a |
| Childhood trauma experience | n (%) | 19 (100) | 6 (100) | 0.999 b |
| Adulthood trauma experience | n (%) | 19 (100) | 6 (100) | 0.999 b |
| **Substance use related measures** |  |  |  |  |
| Severity of Dependence score | Median (IQR) | 11 (10-13) | 14 (11-14) | 0.233 a |
| Drinks to feel any effect lately | Median (IQR) | **3 (2-6)** | **8 (5-11)** | **0.058 a** |
| Drinks to dizzy/slurred speech lately | Median (IQR) | 5 (3-10) | 8 (5-12) | 0.240 a |
| **Blood values** |  |  |  |  |
| g-GT | Median (IQR) | 25 (18-68) | 36 (32-42) | 0.121 |
| Ferritin | Median (IQR) | **99 (41-179)** | **16 (10-25)** | **<0.001** |
| Cholesterol | Median (IQR) | **5.0 (4.7-6.2)** | **4.3 (4.2-)** | **0.044** |

P-values below 0.1 are indicated in bold. BMI: body mass index caculated from height (in meters) and weight (in kg) after the fomula m/kg^2^; HSCL: Hopkins Symptom Cheklist; BDI-II: Beck depression Inventory 2; ASRS: Adult ADHD symptom Rating scale. Descriptive statistics given as medians (IQR (Interquartile range): 25^th^ and 75^th^ percentile) and group differences tested with Mann-Whitney U test for continuous variables (a) and given as frequencies and percent and tested with Fisher exact test for categorical variables (b).

**Supplementary table S2.** Linear regression of the explanatory capability of having undergone bariatric surgery for prediction of Number of units needed to feel any effect of alcohol lately (SRE any effect lately). All p-values below 0.1 are shown in bold.

|  | Beta coefficient | Upper and lower bound  (95% confidence interval) | P-value |
| --- | --- | --- | --- |
| **Number of units needed to feel any effect of alcohol lately** | | |  |
| Crude | **6.4** | **1.7-11.1** | **0.009** |
| Adjusted for gender | **8.3** | **3.5-13.2** | **0.001** |
| Adjusted for gender and BMI | **7.3** | **2.3-12-3** | **0.005** |
| Adjusted for gender, BMI, and HSCL-10 | **7.0** | **2.0-12.1** | **0.007** |
| Adjusted for gender, BMI, HSCL-10, and SDS | **7.1** | **2.0-12.2** | **0.007** |
